# Awareness and Perception Toward Obesity and Knee Osteoarthritis and Their Preventive Measures Among the Adult Population in the Northern Borders Region, Saudi Arabia

**DOI:** 10.7759/cureus.54164

**Published:** 2024-02-14

**Authors:** Haider O Elmisbah, Salah K Almotrafi, Rakan M Alanazi, Fahad M Althagafi, Khalid A Almtuairi, Zakariya M Mohammed

**Affiliations:** 1 Department of Surgery, Faculty of Medicine, Northern Border University, Arar, SAU; 2 Department of Mathematics, College of Science, Northern Border University, Arar, SAU

**Keywords:** knee osteoarthritis, treatment options, preventive measures, health education, saudi arabia, demographics, perceptions, awareness

## Abstract

Background: Osteoarthritis (OA) of the knee poses a significant public health challenge, with its prevalence escalating globally. This study addresses a critical knowledge gap by investigating the awareness and perceptions of knee OA in the Northern Borders Region, Saudi Arabia, focusing on demographic factors that may influence community perspectives.

Study aim: The primary aim of this cross-sectional study is to comprehensively examine the awareness and perceptions of knee OA, exploring the influence of demographic variables, including region, gender, age, nationality, and educational levels.

Methodology: A total of 501 participants from various cities in the Northern Borders Region, Saudi Arabia, were enrolled in this study. Demographic characteristics, including region, gender, age, nationality, and educational levels, were documented. A structured survey instrument was utilized to collect data on awareness and perceptions of knee OA. Statistical analyses included descriptive statistics, chi-square tests, and logistic regression to explore associations.

Results: Demographic insights revealed a predominance of participants from Arar (37.50%) and Rafha (36.50%), with a nearly equal gender distribution (52.90% male, 47.10% female). The majority fell within the 31-45 age group (37.50%), and 97.60% were Saudi nationals. Educational levels varied, with 55.30% holding a bachelor's degree. Awareness levels indicated that 75.40% recognized obesity as a significant factor in knee OA. Significant associations were found between gender and acknowledgment of obesity (p = 0.021), as well as between age and awareness of obesity (p = 0.040). Non-Saudi participants exhibited a higher awareness of knee injury as a reason for arthritis (p = 0.028). Educational levels demonstrated significant associations with awareness of rheumatoid arthritis (p = 0.012), growing old as a reason for knee arthritis (p = 0.002), and various preventive measures and treatment options.

Conclusion: This study provides a nuanced understanding of knee OA awareness and perceptions in the Northern Borders Region, Saudi Arabia. The high recognition of obesity as a risk factor, coupled with demographic variations, highlights the need for tailored health education interventions. Addressing gender-specific, age-related, and educational disparities is crucial for promoting effective community-wide initiatives to prevent and manage knee OA.

## Introduction

Osteoarthritis (OA) is a prevalent and debilitating joint disorder characterized by the progressive degradation of articular cartilage, changes in subchondral bone, and alterations in joint tissues, leading to pain, stiffness, and functional limitations [1]. As a leading cause of musculoskeletal disability worldwide, knee OA poses a substantial public health burden, affecting the quality of life for millions of individuals [2,3].

Like many other regions globally, the Northern Borders Region in Saudi Arabia faces the growing challenge of an aging population and an increased prevalence of chronic diseases [4]. Aging is a significant risk factor for knee OA, and as life expectancy rises, the burden of this condition is anticipated to escalate. Furthermore, lifestyle changes, including sedentary behavior, unhealthy dietary patterns, and an increasing prevalence of obesity, contribute to the rising incidence of knee OA [5,6]. The intersection of these factors underscores the need for comprehensive research and public health initiatives to address knee OA awareness and perceptions among diverse populations [7].

Understanding the awareness and perceptions of knee OA is crucial for developing effective prevention and management strategies. Patient knowledge and beliefs play a pivotal role in healthcare-seeking behavior, adherence to treatment plans, and overall health outcomes [8]. In the context of knee OA, where lifestyle modifications and early interventions can significantly impact disease progression, addressing community awareness becomes a paramount public health priority [9,10].

Despite the recognized importance of knee OA awareness, there is a dearth of research focusing on specific regional populations, such as the Northern Borders Region in Saudi Arabia. Regional variations in demographics, cultural norms, and healthcare accessibility can influence the prevalence of risk factors and the community's understanding of knee OA [11-13]. Tailoring interventions to address these regional nuances is essential for achieving meaningful impact and reducing the burden of knee OA in the Northern Borders Region [7,8]. Moreover, cultural beliefs and societal norms may shape individuals' perceptions about joint disorders, affecting their willingness to seek medical advice, adhere to prescribed treatments, or consider surgical interventions [5]. The multifaceted nature of knee OA, involving both biological and sociocultural determinants, necessitates a comprehensive exploration of community attitudes and knowledge to inform targeted educational and preventive efforts [3,6,8].

Study rationale

In the Northern Borders Region, limited research has been conducted to investigate the awareness and perceptions of knee OA. Existing studies often focus on urban centers, potentially overlooking the unique characteristics and challenges faced by individuals in more rural or less economically developed areas [11,12]. A thorough understanding of the region's sociodemographic landscape, prevalence of risk factors, and community attitudes towards knee OA is crucial for developing context-specific interventions.

As healthcare systems strive to implement evidence-based practices and community-centered interventions, a comprehensive understanding of knee OA awareness and perceptions is instrumental [3,5,8]. This research contributes insights that can guide healthcare providers, policymakers, and public health professionals in developing strategies to enhance community awareness, promote preventive measures, and improve the overall management of knee OA in the unique context of the Northern Borders Region, Saudi Arabia.

Study objectives

Assess the community's awareness and perceptions of knee OA and obesity, explore variations based on demographic factors, and identify the key determinants influencing knowledge and beliefs. Investigate the factors influencing individuals' decisions regarding treatment modalities for knee OA, with a specific focus on the acceptance and apprehensions associated with knee replacement surgery.

## Materials and methods

Study design and setting

This study employed a cross-sectional research design to investigate knee OA awareness and perceptions among participants in the Northern Borders Region, Saudi Arabia. The Northern Borders Region was chosen as the study setting due to its diverse demographic composition and relevance to the research objectives.

Study participants

The sample size was calculated by using the formula N=z² p (1- p) / d² where N = sample size, Z = the statistic corresponding to confidence level (1.96), p = the expected prevalence of awareness (50%), and d = precision (0.05). The expected sample size was 383.

The study included a total of 501 participants selected through a stratified random sampling technique. Stratification was based on the distribution of the population across different cities within the Northern Borders Region. The participants were recruited from Arar, Rafha, Turaif, Oweigeila, and other northern cities, ensuring a representative sample reflecting the region's heterogeneity.

Data collection participants were approached through various community centers, healthcare facilities, and educational institutions. The research team explained the study's purpose, procedures, and confidentiality measures to potential participants. Informed consent was obtained from each participant before their inclusion in the study.

Structured interviews were conducted using a pre-tested questionnaire developed by the research team. The questionnaire comprised sections on demographic information, awareness of knee OA and related risk factors, perceptions about preventive measures and treatment options, and factors influencing the decision to undergo knee replacement surgery.

Demographic variables

Demographic information collected included age, gender, nationality, educational level, city of residence, and the presence of any chronic diseases. These variables were essential for characterizing the study population and exploring potential variations in awareness and perceptions based on demographic factors.

Awareness and perception assessment

The questionnaire included items assessing participants' awareness of knee OA risk factors, preventive measures, and treatment options. Specific questions focused on identifying factors perceived as likely causes of knee arthritis, knowledge of preventive measures, recognition of symptoms, beliefs about curability, and attitudes toward knee replacement surgery.

Data analysis

Data analysis was conducted using the software Statistical Package for Social Sciences (SPSS), version 26.0 (IBM Corp., Armonk, NY), employing both descriptive and inferential statistical methods. Descriptive statistics, including frequencies and percentages, were computed for demographic variables, awareness, and perception items. Inferential statistics, such as chi-square tests, were used to explore associations between demographic variables and awareness and perception outcomes. Significance was set at a p-value of less than 0.05.

Ethical considerations

This study adhered to the ethical principles outlined in the Declaration of Helsinki. Ethical approval was obtained from the Committee of Bioethics at Northern Border University (HAP-09-A-043) which has issued approval No. 54/44/H dated July 5, 2023. Informed consent was prioritized, ensuring participants had a clear understanding of the study's purpose, procedures, and their rights to voluntary participation and withdrawal.

Inclusion and exclusion criteria

Men and women, Arabic speaking, and who reside in the Northern Borders Region were included. Those who suffer from OA, non-Arabic speaking, and those who reside outside the Northern Borders Region were excluded from the study.

## Results

In Table 1, the characteristics of the included participants (n = 501) are presented. Regarding the distribution by region, the majority of participants were from Arar, constituting 37.50% (n = 188) of the total sample. Rafha closely followed, representing 36.50% (n = 183). Turaif and Oweigeila each accounted for 2.80% (n = 14), while 20.40% (n = 102) were from other northern cities. The gender distribution of the participants is also noteworthy, with 52.90% (n = 265) being male and 47.10% (n = 236) being female.

**Table 1:** Characteristics of the included participants (n = 501).

Parameter		Frequency (%)
Region	Arar	188 (37.50%)
	Rafha	183 (36.50%)
	Turaif	14 (2.80%)
	Oweigeila	14 (2.80%)
	Other Northern cities	102 (20.40%)
Sex	Male	265 (52.90%)
	Female	236 (47.10%)
Age	<18	21 (4.20%)
	18-30	186 (37.10%)
	31-45	188 (37.50%)
	46-65	102 (20.40%)
	>=65	4 (0.80%)
Nationality	Saudi	489 (97.60%)
	Non-Saudi	12 (2.40%)
Education level	Illiterate	2 (0.40%)
	Primary or intermediate	23 (4.60%)
	Secondary	97 (19.40%)
	Diploma	86 (17.20%)
	Bachelor	277 (55.30%)
	Postgraduate	16 (3.20%)
Chronic diseases	Hypertension	37 (7.40%)
	Diabetes	43 (8.60%)
	Bronchial asthma	20 (4%)
	I don't suffer	395 (78.80%)
	Others	20 (4%)

Age-wise categorization reveals a varied representation of participants. The age group of 31-45 constitutes the largest portion, representing 37.50% (n = 188), followed closely by the 18-30 age group at 37.10% (n = 186). In terms of nationality, the majority of participants were Saudi nationals, comprising 97.6% (n = 489), while 2.40% (n = 12) were non-Saudi. Educational levels among participants varied, with the majority holding a bachelor's degree (55.30%, n = 277), followed by secondary education (19.40%, n = 97). The distribution of participants with chronic diseases provides additional context. Hypertension was reported by 7.40% (n = 37), diabetes by 8.60% (n = 43), bronchial asthma by 4% (n = 20), and 78.80% (n = 395) stated that they do not suffer from any chronic diseases.

Table 2 provides a comprehensive overview of the awareness and perceptions of the 501 participants toward knee OA and obesity. Regarding the likely reasons for knee arthritis, the majority of participants identified obesity as a significant factor, with 75.40% (n = 378) acknowledging its role. Repeated pressure on the knee was also recognized by 36.30% (n = 182) of participants. Interestingly, only 8.40% (n = 42) considered the patient's sex as a likely reason, emphasizing awareness of more prominent factors such as obesity and pressure on the knee.

**Table 2:** Awareness and perception of participants toward knee OA and obesity (n = 501).

Parameter		Yes	No
Likely, reasons for knee arthritis?	Obesity	378 (75.40%)	123 (24.60%)
	Repeated pressure on the knee	182 (36.30%)	319 (63.70%)
	Patient's sex	42 (8.40%)	459 (91.60%)
	Knee injury	149 (29.70%)	352 (70.30%)
	Lack of movement	196 (39.10%)	305 (60.90%)
	Genetic reasons	71 (14.20%)	430 (85.80%)
	Rheumatoid arthritis	204 (40.70%)	297 (59.30%)
	Growing old	209 (41.70%)	292 (58.30%)
	Wrong sitting positions	214 (42.70%)	287 (57.30%)
In your opinion, what are the preventive measures that prevent the risk of developing knee arthritis?	Lose weight	377 (75.20%)	124 (24.80%)
	Adhere to the correct sitting positions	234 (46.70%)	267 (53.30%)
	Avoid constant pressure on the knee	205 (40.90%)	296 (59.10%)
	Exercise and motor activity	320 (63.90%)	181 (36.10%)
What do you think are the most prominent symptoms of knee arthritis?	Knee pain	354 (70.70%)	147 (29.30%)
	Knee swelling	184 (36.70%)	317 (63.30%)
	Curvature of the legs	56 (11.20%)	445 (88.80%)
	Knee joint stiffness	184 (36.70%)	317 (63.30%)
	Popping sound during movement	212 (42.30%)	289 (57.70%)
In your opinion	Is there a cure for knee arthritis?	464 (92.60%)	37 (7.40%)
In your opinion, what are the methods that help in the treatment of arthritis of the knee?	Exercise	267 (53.30%)	234 (46.70%)
	Hot compresses	76 (15.20%)	425 (84.80%)
	Cold compresses	66 (13.20%)	435 (86.80%)
	Topical cortisone injections	121 (24.20%)	380 (75.80%)
	Drugs	136 (27.10%)	365 (72.90%)
	Physical therapy	295 (58.90%)	206 (41.10%)
	Surgical intervention	138 (27.50%)	363 (72.50%)
	Lose weight	292 (58.30%)	209 (41.70%)
In your opinion, what is the purpose of knee replacement surgery?	Relieve pain	355 (70.90%)	146 (29.10%)
	Increase walking quality	244 (48.70%)	257 (51.30%)
	Ability to exercise	170 (33.90%)	331 (66.10%)
	Ability to perform prayer	251 (50.10%)	250 (49.90%)
What are the reasons that may prevent you from undergoing knee replacement surgery if you need it?	Postoperative pain	265 (52.90%)	236 (47.10%)
	General anesthesia and its complications	167 (33.30%)	334 (66.70%)
	Surgery is not helpful	112 (22.40%)	389 (77.60%)
	Surgeons are not available	145 (28.90%)	356 (71.10%)
Do you think surgery is the best treatment if non-surgical options don't work?		434 (86.60%)	67 (13.40%)
Do you think there is a link between knee arthritis and obesity?		472 (94.20%)	29 (5.80%)

In terms of preventive measures, a substantial proportion of participants recognized the importance of losing weight (75.20%, n = 377) and adhering to correct sitting positions (46.70%, n = 234). The identification of prominent symptoms of knee arthritis revealed that 70.70% (n = 354) recognized knee pain as a significant indicator, while knee swelling, knee joint stiffness, and popping sounds during movement were also acknowledged by substantial percentages of participants.

A noteworthy finding is that 92.60% (n = 464) of participants believe there is a cure for knee arthritis. Concerning treatment methods, a considerable proportion of participants endorsed exercise (53.30%, n = 267) and physical therapy (58.90%, n = 295) as effective interventions.

When considering knee replacement surgery, the majority of participants associated it with pain relief (70.90%, n = 355) and an improvement in walking quality (48.70%, n = 244). Notably, the ability to perform prayer was considered a relevant outcome by 50.10% (n = 251) of participants, highlighting the multifaceted expectations associated with knee replacement surgery.

The participants' perceptions of factors that may prevent them from undergoing knee replacement surgery demonstrated concerns related to postoperative pain (52.90%, n = 265) and general anesthesia complications (33.30%, n = 167). Additionally, 22.40% (n = 112) expressed doubts about the effectiveness of the surgery itself.

Table 3 presents a detailed analysis of the association between sex and the awareness and perception of 501 participants toward knee OA and obesity. In examining the reasons for knee arthritis, a significant association was found between sex and the acknowledgment of obesity as a contributing factor (p-value = 0.021). Specifically, 79.60% of males and 70.80% of females recognized obesity as a likely cause.

**Table 3:** Sex in association with awareness and perception of participants toward knee OA and obesity (n = 501). * The Chi-square statistic is significant at the 0.05 level. OA: Osteoarthritis.

Parameter		Sex		P-value
		Male	Female	
Reasons for knee arthritis				
Obesity	Yes	211 (79.60%)	167 (70.80%)	0.021^*^
	No	54 (20.40%)	69 (29.20%)	
Repeated pressure on the knee	Yes	99 (37.40%)	83 (35.20%)	0.611
	No	166 (62.60%)	153 (64.80%)	
The patient's sex	Yes	32 (12.10%)	10 (4.20%)	0.002^*^
	No	233 (87.90%)	226 (95.80%)	
knee injury	Yes	76 (28.70%)	73 (30.90%)	0.582
	No	189 (71.30%)	163 (69.10%)	
Lack of movement	Yes	98 (37%)	98 (41.50%)	0.298
	No	167 (63%)	138 (58.50%)	
Genetic reasons	Yes	42 (15.80%)	29 (12.30%)	0.254
	No	223 (84.20%)	207 (87.70%)	
Rheumatoid arthritis	Yes	102 (38.50%)	102 (43.20%)	0.282
	No	163 (61.50%)	134 (56.80%)	
Growing old	Yes	119 (44.90%)	90 (38.1%)	0.125
	No	146 (55.10%)	146 (61.90%)	
Wrong sitting positions	Yes	116 (43.80%)	98 (41.50%)	0.612
	No	149 (56.20%)	138 (58.50%)	
Prevention measures for knee arthritis				
Lose weight	Yes	205 (77.40%)	172 (72.90%)	0.246
	No	60 (22.60%)	64 (27.10%)	
Adhere to the correct sitting positions	Yes	123 (46.40%)	111 (47%)	0.890
	No	142 (53.60%)	125 (53%)	
Avoid constant pressure on the knee	Yes	114 (43%)	91 (38.60%)	0.311
	No	151 (57%)	145 (61.40%)	
Exercise and motor activity	Yes	167 (63%)	153 (64.80%)	
	No	98 (37%)	83 (35.20%)	
Methods helping in the treatment of knee OA				
Exercise	Yes	128 (48.30%)	139 (58.90%)	0.018^*^
	No	137 (51.70%)	97 (41.10%)	
hot compresses	Yes	33 (12.50%)	43 (18.20%)	0.072
	No	232 (87.50%)	193 (81.80%)	
Cold compresses	Yes	44 (16.60%)	22 (9.30%)	0.016^*^
	No	221 (83.40%)	214 (90.70%)	
Topical cortisone injections	Yes	66 (24.90%)	55 (23.30%)	0.676
	No	199 (75.10%)	181 (76.70%)	
Drugs	Yes	80 (30.20%)	56 (23.70%)	0.105
	No	185 (69.80%)	180 (76.30%)	
Physical therapy	Yes	163 (61.50%)	132 (55.90%)	0.205
	No	102 (38.50%)	104 (44.10%)	
Surgical intervention	Yes	94 (35.50%)	44 (18.60%)	0.000^*^
	No	171 (64.50%)	192 (81.40%)	
Lose weight	Yes	164 (61.90%)	128 (54.20%)	0.083
	No	101 (38.10%)	108 (45.80%)	
Purpose of knee replacement surgery				
Relieve pain	Yes	198 (74.70%)	157 (66.50%)	0.044^*^
	No	67 (25.30%)	79 (33.50%)	
Increase walking quality	Yes	115 (43.40%)	129 (54.70%)	0.012^*^
	No	150 (56.60%)	107 (45.30%)	
The ability to exercise	Yes	108 (40.80%)	62 (26.30%)	0.001^*^
	No	157 (59.20%)	174 (73.70%)	
The ability to perform prayer	Yes	141 (53.20%)	110 (46.60%)	0.140
	No	124 (46.8%)	126 (53.40%)	
Reasons preventing you from undergoing knee replacement surgery				
Postoperative pain	Yes	136 (51.30%)	129 (54.70%)	0.455
	No	129 (48.70%)	107 (45.30%)	
General anesthesia and its complications	Yes	89 (33.60%)	78 (33.10%)	0.899
	No	176 (66.40%)	158 (66.90%)	
Surgery is not helpful	Yes	60 (22.60%)	52 (22%)	0.871
	No	205 (77.40%)	184 (78%)	
Surgeons are not available	Yes	91 (34.30%)	54 (22.90%)	0.005^*^
	No	174 (65.70%)	182 (77.10%)	

When assessing preventive measures, the association between sex and the endorsement of exercise as a preventive measure for knee arthritis was statistically significant (p-value = 0.018). Notably, 48.30% of males, compared to 58.90% of females, identified exercise as a preventive measure.

The analysis of methods helping in the treatment of knee OA revealed several noteworthy associations. Females showed a higher recognition of the effectiveness of cold compresses (16.60% vs. 9.30%, p-value = 0.016) and a preference for surgical intervention (35.50% vs. 18.60%, p-value = 0.000) compared to males.

In assessing the purpose of knee replacement surgery, a statistically significant association was observed between sex and the perceived relief of pain (74.70% of males vs. 66.50% of females, p-value = 0.044), increased walking quality (43.40% of males vs. 54.70% of females, p-value = 0.012), and the ability to exercise (40.80% of males vs. 26.30% of females, p-value = 0.001). Notably, the decision-making process for undergoing knee replacement surgery was significantly influenced by the availability of surgeons (34.30% of males vs. 22.90% of females, p-value = 0.005).

Table 4 presents a comprehensive examination of the association between age groups and the awareness and perception of 501 participants toward knee OA and obesity. In investigating the reasons for knee arthritis, a significant association was found between age and the acknowledgment of obesity as a contributing factor (p-value = 0.040). Participants aged 46-65 years showed the highest awareness, with 78.70% recognizing obesity as a likely cause, while those aged <18 years exhibited the lowest awareness at 57.10%.

**Table 4:** Age in association with awareness and perception of participants toward knee OA and obesity (n=501). * The Chi-square statistic is significant at the 0.05 level. OA: Osteoarthritis.

Parameter		Age					P-value
		<18	18-30	31-45	46-65	>=65	
Reasons for knee arthritis							
Obesity	Yes	12 (57.10%)	131 (70.40%)	148 (78.70%)	84 (82.40%)	3 (75%)	0.040^*^
	No	9 (42.90%)	55 (29.60%)	40 (21.30%)	18 (17.60%)	1 (25%)	
Repeated pressure on the knee	Yes	8 (38.10%)	81 (43.50%)	70 (37.20%)	21 (20.60%)	2 (50%)	0.004^*^
	No	13 (61.90%)	105 (56.50%)	118 (62.80%)	81 (79.40%)	2 (50%)	
The patient's sex	Yes	1 (4.80%)	22 (11.80%)	10 (5.30%)	9 (8.80%)	0 (0%)	0.205
	No	20 (95.20%)	164 (88.20%)	178 (94.70%)	93 (91.20%)	4 (100%)	
Knee injury	Yes	6 (28.60%)	63 (33.90%)	57 (30.30%)	23 (22.50%)	0 (0%)	0.216
	No	15 (71.40%)	123 (66.10%)	131 (69.70%)	79 (77.50%)	4 (100%)	
Lack of movement	Yes	10 (47.60%)	69 (37.10%)	78 (41.50%)	38 (37.30%)	1 (25%)	0.757
	No	11 (52.40%)	117 (62.90%)	110 (58.50%)	64 (62.70%)	3 (75%)	
Genetic reasons	Yes	6 (28.60%)	34 (18.30%)	15 (8%)	15 (14.70%)	1 (25%)	0.014^*^
	No	15 (71.40%)	152 (81.70%)	173 (92%)	87 (85.30%)	3 (75%)	
Rheumatoid arthritis	Yes	11 (52.40%)	78 (41.90%)	76 (40.40%)	38 (37.30%)	1 (25%)	0.695
	No	10 (47.60%)	108 (58.10%)	112 (59.60%)	64 (62.70%)	3 (75%)	
Growing old	Yes	8 (38.10%)	84 (45.20%)	74 (39.40%)	40 (39.20%)	3 (75%)	0.473
	No	13 (61.90%)	102 (54.80%)	114 (60.60%)	62 (60.80%)	1 (25%)	
Wrong sitting positions	Yes	8 (38.10%)	80 (43%)	85 (45.20%)	39 (38.20%)	2 (50%)	0.81
	No	13 (61.90%)	106 (57%)	103 (54.80%)	63 (61.80%)	2 (50%)	
Prevention measures for knee arthritis							
Lose weight	Yes	11 (52.40%)	131 (70.40%)	149 (79.30%)	83 (81.40%)	3 (75%)	0.018^*^
	No	10 (47.60%)	55 (29.60%)	39 (20.70%)	19 (18.60%)	1 (25%)	
Adhere to the correct sitting positions	Yes	11 (52.40%)	90 (48.40%)	93 (49.50%)	36 (35.30%)	4 (100%)	0.027^*^
	No	10 (47.60%)	96 (51.60%)	95 (50.50%)	66 (64.70%)	0 (0%)	
Avoid constant pressure on the knee	Yes	9 (42.90%)	83 (44.60%)	83 (44.10%)	29 (28.40%)	1 (25%)	0.064
	No	12 (57.10%)	103 (55.40%)	105 (55.90%)	73 (71.60%)	3 (75%)	
Exercise and motor activity	Yes	17 (81%)	123 (66.10%)	122 (64.90%)	56 (54.90%)	2 (50%)	0.134
	No	4 (19%)	63 (33.90%)	66 (35.10%)	46 (45.10%)	2 (50%)	
Methods helping in the treatment of knee OA							
Exercise	Yes	15 (71.40%)	101 (54.30%)	113 (60.10%)	37 (36.30%)	1 (25%)	0.001^*^
	No	6 (28.60%)	85 (45.70%)	75 (39.90%)	65 (63.70%)	3 (75%)	
Hot compresses	Yes	3 (14.30%)	25 (13.40%)	31 (16.50%)	16 (15.70%)	1 (25%)	0.907
	No	18 (85.70%)	161 (86.60%)	157 (83.5%)	86 (84.30%)	3 (75%)	
Cold compresses	Yes	1 (4.80%)	31 (16.70%)	25 (13.30%)	9 (8.80%)	0 (0%)	0.233
	No	20 (95.20%)	155 (83.30%)	163 (86.70%)	93 (91.20%)	4 (100%)	
Topical cortisone injections	Yes	2 (9.50%)	47 (25.30%)	47 (25%)	23 (22.50%)	2 (50%)	0.373
	No	19 (90.50%)	139 (74.70%)	141 (75%)	79 (77.50%)	2 (50%)	
Drugs	Yes	6 (28.60%)	58 (31.20%)	49 (26.10%)	22 (21.60%)	1 (25%)	0.512
	No	15 (71.40%)	128 (68.80%)	139 (73.90%)	80 (78.40%)	3 (75%)	
Physical therapy	Yes	13 (61.90%)	117 (62.90%)	110 (58.50%)	53 (52%)	2 (50%)	0.481
	No	8 (38.10%)	69 (37.10%)	78 (41.50%)	49 (48%)	2 (50%)	
Surgical intervention	Yes	2 (9.50%)	52 (28%)	44 (23.40%)	38 (37.30%)	2 (50%)	0.028^*^
	No	19 (90.50%)	134 (72%)	144 (76.60%)	64 (62.70%)	2 (50%)	
Lose weight	Yes	11 (52.40%)	92 (49.50%)	114 (60.60%)	71 (69.60%)	4 (100%)	0.005^*^
	No	10 (47.60%)	94 (50.50%)	74 (39.40%)	31 (30.40%)	0 (0%)	
Purpose of knee replacement surgery							
Relieve pain	Yes	12 (57.10%)	129 (69.40%)	128 (68.10%)	84 (82.40%)	2 (50%)	0.037^*^
	No	9 (42.90%)	57 (30.60%)	60 (31.90%)	18 (17.60%)	2 (50%)	
Increase walking quality	Yes	11 (52.40%)	86 (46.20%)	104 (55.30%)	41 (40.20%)	2 (50%)	0.146
	No	10 (47.60%)	100 (53.80%)	84 (44.70%)	61 (59.80%)	2 (50%)	
The ability to exercise	Yes	9 (42.90%)	75 (40.30%)	62 (33%)	22 (21.60%)	2 (50%)	0.020^*^
	No	12 (57.10%)	111 (59.70%)	126 (67%)	80 (78.40%)	2 (50%)	
The ability to perform prayer	Yes	12 (57.10%)	85 (45.70%)	97 (51.60%)	54 (52.90%)	3 (75%)	0.501
	No	9 (42.90%)	101 (54.30%)	91 (48.40%)	48 (47.10%)	1 (25%)	
Reasons preventing you fromr undergoing knee replacement surgery							
Postoperative pain	Yes	10 (47.60%)	98 (52.70%)	102 (54.30%)	54 (52.90%)	1 (25%)	0.804
	No	11 (52.40%)	88 (47.30%)	86 (45.70%)	48 (47.10%)	3 (75%)	
General anesthesia and its complications	Yes	8 (38.10%)	78 (41.90%)	58 (30.90%)	22 (21.60%)	1 (25%)	0.009^*^
	No	13 (61.90%)	108 (58.10%)	130 (69.10%)	80 (78.40%)	3 (75%)	
Surgery is not helpful	Yes	7 (33.30%)	37 (19.90%)	43 (22.90%)	23 (22.50%)	2 (50%)	0.42
	No	14 (66.70%)	149 (80.10%)	145 (77.10%)	79 (77.50%)	2 (50%)	
Surgeons are not available	Yes	5 (23.80%)	45 (24.20%)	58 (30.90%)	34 (33.30%)	3 (75%)	0.102
	No	16 (76.20%)	141 (75.80%)	130 (69.10%)	68 (66.70%)	1 (25%)	

For repeated pressure on the knee, a statistically significant association was observed with age (p-value = 0.004). Participants under 18 years old demonstrated the lowest awareness (38.10%), while those aged 46-65 years exhibited the highest awareness (20.60%). Regarding genetic reasons for knee arthritis, a significant association with age was identified (p-value = 0.014). Participants under 18 years old showed the lowest awareness (28.60%), while those aged 31-45 years exhibited the highest awareness (92%).

In examining preventive measures, a significant association with age was found for the endorsement of losing weight as a preventive measure (p-value = 0.018). Participants aged 46-65 years demonstrated the highest awareness (79.30%), while those under 18 years exhibited the lowest awareness (52.40%).

The association between age and the recognition of exercise as a method helping in the treatment of knee OA was statistically significant (p-value = 0.001). Participants under 18 years old showed the lowest awareness (71.40%), while those aged 46-65 years exhibited the highest awareness (36.30%). Regarding the purpose of knee replacement surgery, a significant association with age was found for relieving pain (p-value = 0.037), the ability to exercise (p-value = 0.020), and undergoing surgical intervention (p-value = 0.028). Participants aged 46-65 years consistently exhibited the highest awareness across these categories.

Table 5 presents a detailed analysis of the association between nationality and the awareness and perception of 501 participants toward knee OA and obesity. In assessing the reasons for knee arthritis, no statistically significant association was observed between nationality and the acknowledgment of obesity as a contributing factor (p-value = 0.971). Both Saudi and non-Saudi participants demonstrated similar awareness levels, with 75.50% and 75%, respectively. For knee injury as a reason for arthritis, a statistically significant association was found with nationality (p-value = 0.028). Non-Saudi participants exhibited a higher level of awareness (58.30%) compared to Saudi participants (29%). No significant associations were observed between nationality and awareness of other reasons for knee arthritis, such as repeated pressure on the knee, the patient's sex, lack of movement, genetic reasons, rheumatoid arthritis, growing old, and wrong sitting positions.

**Table 5:** Nationality in association with awareness and perception of participants toward knee OA and obesity (n=501). * The Chi-square statistic is significant at the 0.05 level. OA: Osteoarthritis.

Parameter		Nationality		P-value
		Saudi	Non-Saudi	
Reasons for knee arthritis				
Obesity	Yes	369 (75.50%)	9 (75%)	0.971
	No	120 (24.50%)	3 (25%)	
Repeated pressure on the knee	Yes	178 (36.40%)	4 (33.30%)	0.827
	No	311 (63.60%)	8 (66.70%)	
The patient's sex	Yes	41 (8.40%)	1 (8.30%)	0.995
	No	448 (91.60%)	11 (91.70%)	
Knee injury	Yes	142 (29%)	7 (58.30%)	0.028^*^
	No	347 (71%)	5 (41.70%)	
Lack of movement	Yes	192 (39.30%)	4 (33.30%)	0.677
	No	297 (60.70%)	8 (66.70%)	
Genetic reasons	Yes	71 (14.50%)	0 (0%)	0.154
	No	418 (85.50%)	12 (100%)	
Rheumatoid arthritis	Yes	200 (40.90%)	4 (33.30%)	0.598
	No	289 (59.10%)	8 (66.70%)	
Growing old	Yes	206 (42.10%)	3 (25%)	0.235
	No	283 (57.90%)	9 (75%)	
Wrong sitting positions	Yes	210 (42.90%)	4 (33.30%)	0.506
	No	279 (57.10%)	8 (66.70%)	
Prevention measures for knee arthritis				
Lose weight	Yes	370 (75.70%)	7 (58.30%)	0.169
	No	119 (24.30%)	5 (41.70%)	
Adhere to the correct sitting positions	Yes	228 (46.60%)	6 (50%)	0.817
	No	261 (53.40%)	6 (50%)	
Avoid constant pressure on the knee	Yes	198 (40.50%)	7 (58.30%)	0.214
	No	291 (59.50%)	5 (41.70%)	
Exercise and motor activity	Yes	311 (63.60%)	9 (75%)	0.417
	No	178 (36.40%)	3 (25%)	
Methods helping in the treatment of knee OA				
Exercise	Yes	261 (53.40%)	6 (50%)	0.817
	No	228 (46.60%)	6 (50%)	
Hot compresses	Yes	74 (15.10%)	2 (16.70%)	0.884
	No	415 (84.90%)	10 (83.30%)	
Cold compresses	Yes	65 (13.30%)	1 (8.30%)	0.616
	No	424 (86.70%)	11 (91.70%)	
Topical cortisone injections	Yes	116 (23.70%)	5 (41.70%)	0.151
	No	373 (76.30%)	7 (58.30%)	
Drugs	Yes	134 (27.40%)	2 (16.70%)	0.409
	No	355 (72.60%)	10 (83.30%)	
Physical therapy	Yes	287 (58.70%)	8 (66.70%)	0.579
	No	202 (41.30%)	4 (33.30%)	
Surgical intervention	Yes	136 (27.80%)	2 (16.70%)	0.393
	No	353 (72.20%)	10 (83.30%)	
Lose weight	Yes	283 (57.90%)	9 (75%)	0.235
	No	206 (42.10%)	3 (25%)	
Purpose of knee replacement surgery				
Relieve pain	Yes	348 (71.20%)	7 (58.30%)	0.334
	No	141 (28.80%)	5 (41.70%)	
Increase walking quality	Yes	238 (48.70%)	6 (50%)	0.927
	No	251 (51.30%)	6 (50%)	
The ability to exercise	Yes	167 (34.20%)	3 (25%)	0.508
	No	322 (65.80%)	9 (75%)	
The ability to perform prayer	Yes	245 (50.10%)	6 (50%)	0.994
	No	244 (49.90%)	6 (50%)	
Reasons preventing you from undergoing knee replacement surgery				
Postoperative pain	Yes	260 (53.20%)	5 (41.70%)	0.430
	No	229 (46.80%)	7 (58.30%)	
General anesthesia and its complications	Yes	165 (33.70%)	2 (16.70%)	0.215
	No	324 (66.30%)	10 (83.30%)	
Surgery is not helpful	Yes	107 (21.90%)	5 (41.70%)	0.104
	No	382 (78.10%)	7 (58.30%)	
Surgeons are not available	Yes	142 (29%)	3 (25%)	0.761
	No	347 (71%)	9 (75%)	

Regarding preventive measures for knee arthritis, no statistically significant association was found between nationality and awareness of losing weight (p-value = 0.169), adhering to correct sitting positions (p-value = 0.817), avoiding constant pressure on the knee (p-value = 0.214), and promoting exercise and motor activity (p-value = 0.417). In exploring methods for treating knee OA, no significant associations were observed between nationality and awareness of exercise, hot compresses, cold compresses, topical cortisone injections, drugs, physical therapy, and surgical intervention.

For the purpose of knee replacement surgery, no statistically significant associations were found between nationality and awareness of relieving pain, increasing walking quality, the ability to exercise, and the ability to perform prayer. In examining factors influencing the decision to undergo knee replacement surgery, no significant associations were observed between nationality and awareness of postoperative pain, general anesthesia and its complications, surgery not being helpful, and the availability of surgeons.

Table 6 provides a comprehensive analysis of the association between educational levels and the awareness and perception of 501 participants toward knee OA and obesity. In assessing the reasons for knee arthritis, no statistically significant association was observed between educational levels and the acknowledgment of obesity as a contributing factor (p-value = 0.576). The participants with different educational backgrounds demonstrated relatively similar awareness levels, ranging from 69.10% to 78%, suggesting a consistent understanding of the impact of obesity on knee arthritis across various educational levels.

**Table 6:** Educational level in association with awareness and perception of participants toward knee OA and obesity (n=501). * The Chi-square statistic is significant at the 0.05 level. OA: Osteoarthritis.

Parameter		Educational level						P-value
		No education	Primary or intermediate	Secondary	Diploma	Bachelor	Postgraduate	
Reasons for knee arthritis								
Obesity	Yes	2 (100%)	17 (73.90%)	67 (69.10%)	64 (74.40%)	216 (78%)	12 (75%)	0.576
	No	0 (0%)	6 (26.10%)	30 (30.90%)	22 (25.60%)	61 (22%)	4 (25%)	
Repeated pressure on the knee	Yes	0 (0%)	7 (30.40%)	28 (28.90%)	26 (30.20%)	116 (41.90%)	5 (31.30%)	0.106
	No	2 (100%)	16 (69.60%)	69 (71.10%)	60 (69.80%)	161 (58.10%)	11 (68.80%)	
The patient's sex	Yes	0 (0%)	2 (8.70%)	7 (7.20%)	4 (4.70%)	28 (10.10%)	1 (6.30%)	0.687
	No	2 (100%)	21 (91.30%)	90 (92.80%)	82 (95.30%)	249 (89.90%)	15 (93.80%)	
Knee injury	Yes	0 (0%)	7 (30.40%)	21 (21.60%)	27 (31.40%)	89 (32.10%)	5 (31.30%)	0.444
	No	2 (100%)	16 (69.60%)	76 (78.40%)	59 (68.60%)	188 (67.90%)	11 (68.80%)	
Lack of movement	Yes	0 (0%)	11 (47.80%)	38 (39.20%)	38 (44.20%)	104 (37.50%)	5 (31.30%)	0.601
	No	2 (100%)	12 (52.20%)	59 (60.80%)	48 (55.80%)	173 (62.50%)	11 (68.80%)	
Genetic reasons	Yes	0 (0%)	5 (21.70%)	9 (9.30%)	12 (14%)	44 (15.90%)	1 (6.30%)	0.438
	No	2 (100%)	18 (78.30%)	88 (90.70%)	74 (86%)	233 (84.10%)	15 (93.80%)	
Rheumatoid arthritis	Yes	0 (0%)	8 (34.80%)	28 (28.90%)	29 (33.70%)	131 (47.30%)	8 (50%)	0.012^*^
	No	2 (100%)	15 (65.20%)	69 (71.10%)	57 (66.30%)	146 (52.70%)	8 (50%)	
Growing old	Yes	0 (0%)	7 (30.40%)	26 (26.80%)	34 (39.50%)	137 (49.50%)	5 (31.30%)	0.002^*^
	No	2 (100%)	16 (69.60%)	71 (73.20%)	52 (60.50%)	140 (50.50%)	11 (68.80%)	
Wrong sitting positions	Yes	0 (0%)	9 (39.10%)	32 (33%)	40 (46.50%)	126 (45.50%)	7 (43.80%)	0.24
	No	2 (100%)	14 (60.90%)	65 (67%)	46 (53.50%)	151 (54.50%)	9 (56.30%)	
Prevention measures for knee arthritis								
Lose weight	Yes	2 (100%)	17 (73.90%)	61 (62.90%)	61 (70.90%)	224 (80.90%)	12 (75%)	0.014^*^
	No	0 (0%)	6 (26.10%)	36 (37.10%)	25 (29.10%)	53 (19.10%)	4 (25%)	
Adhere to the correct sitting positions	Yes	0 (0%)	8 (34.80%)	50 (51.50%)	38 (44.20%)	128 (46.20%)	10 (62.50%)	0.323
	No	2 (100%)	15 (65.20%)	47 (48.50%)	48 (55.80%)	149 (53.80%)	6 (37.50%)	
Avoid constant pressure on the knee	Yes	0 (0%)	6 (26.10%)	36 (37.10%)	32 (37.20%)	123 (44.40%)	8 (50%)	0.262
	No	2 (100%)	17 (73.90%)	61 (62.90%)	54 (62.80%)	154 (55.60%)	8 (50%)	
Exercise and motor activity	Yes	0 (0%)	18 (78.30%)	54 (55.70%)	50 (58.10%)	186 (67.10%)	12 (75%)	0.038^*^
	No	2 (100%)	5 (21.70%)	43 (44.30%)	36 (41.90%)	91 (32.90%)	4 (25%)	
Methods helping in the treatment of knee OA								
Exercise	Yes	2 (100%)	15 (65.20%)	46 (47.40%)	45 (52.30%)	149 (53.80%)	10 (62.50%)	0.414
	No	0 (0%)	8 (34.80%)	51 (52.60%)	41 (47.70%)	128 (46.20%)	6 (37.50%)	
Hot compresses	Yes	0 (0%)	3 (13%)	16 (16.50%)	17 (19.80%)	35 (12.60%)	5 (31.30%)	0.254
	No	2 (100%)	20 (87%)	81 (83.50%)	69 (80.20%)	242 (87.40%)	11 (68.80%)	
Cold compresses	Yes	0 (0%)	1 (4.30%)	12 (12.40%)	9 (10.50%)	39 (14.10%)	5 (31.30%)	0.203
	No	2 (100%)	22 (95.70%)	85 (87.60%)	77 (89.50%)	238 (85.90%)	11 (68.80%)	
Topical cortisone injections	Yes	0 (0%)	2 (8.70%)	16 (16.50%)	17 (19.80%)	81 (29.20%)	5 (31.30%)	0.035^*^
	No	2 (100%)	21 (91.3%)	81 (83.5%)	69 (80.2%)	196 (70.8%)	11 (68.8%)	
Drugs	Yes	0 (0%)	6 (26.10%)	25 (25.80%)	14 (16.30%)	81 (29.20%)	10 (62.50%)	0.005^*^
	No	2 (100%)	17 (73.90%)	72 (74.20%)	72 (83.70%)	196 (70.80%)	6 (37.50%)	
Physical therapy	Yes	0 (0%)	13 (56.50%)	50 (51.50%)	43 (50%)	182 (65.70%)	7 (43.80%)	0.012^*^
	No	2 (100%)	10 (43.5%)	47 (48.5%)	43 (50%)	95 (34.3%)	9 (56.3%)	
Surgical intervention	Yes	0 (0%)	2 (8.70%)	21 (21.60%)	20 (23.30%)	92 (33.20%)	3 (18.80%)	0.030^,*^
	No	2 (100%)	21 (91.30%)	76 (78.40%)	66 (76.70%)	185 (66.80%)	13 (81.30%)	
Lose weight	Yes	0 (0%)	14 (60.90%)	54 (55.70%)	41 (47.70%)	172 (62.10%)	11 (68.80%)	0.091
	No	2 (100%)	9 (39.10%)	43 (44.30%)	45 (52.30%)	105 (37.90%)	5 (31.30%)	
Purpose of knee replacement surgery								
Relieve pain	Yes	2 (100%)	16 (69.60%)	63 (64.90%)	60 (69.80%)	201 (72.60%)	13 (81.30%)	0.585
	No	0 (0%)	7 (30.40%)	34 (35.10%)	26 (30.20%)	76 (27.40%)	3 (18.80%)	
Increase walking quality	Yes	0 (0%)	11 (47.80%)	37 (38.10%)	41 (47.70%)	148 (53.40%)	7 (43.80%)	0.113
	No	2 (100%)	12 (52.20%)	60 (61.90%)	45 (52.30%)	129 (46.60%)	9 (56.30%)	
The ability to exercise	Yes	0 (0%)	10 (43.50%)	34 (35.10%)	25 (29.10%)	96 (34.70%)	5 (31.30%)	0.694
	No	2 (100%)	13 (56.50%)	63 (64.90%)	61 (70.90%)	181 (65.30%)	11 (68.80%)	
The ability to perform prayer	Yes	0 (0%)	12 (52.20%)	49 (50.50%)	43 (50%)	137 (49.50%)	10 (62.50%)	0.687
	No	2 (100%)	11 (47.80%)	48 (49.50%)	43 (50%)	140 (50.50%)	6 (37.50%)	
Reasons preventing you for undergoing knee replacement surgery								
Postoperative pain	Yes	1 (50%)	14 (60.90%)	47 (48.50%)	44 (51.20%)	149 (53.80%)	10 (62.50%)	0.828
	No	1 (50%)	9 (39.10%)	50 (51.50%)	42 (48.80%)	128 (46.20%)	6 (37.50%)	
General anesthesia and its complications	Yes	1 (50%)	8 (34.80%)	32 (33%)	25 (29.10%)	94 (33.90%)	7 (43.80%)	0.875
	No	1 (50%)	15 (65.20%)	65 (67%)	61 (70.90%)	183 (66.10%)	9 (56.30%)	
Surgery is not helpful	Yes	0 (0%)	7 (30.40%)	22 (22.70%)	17 (19.80%)	66 (23.80%)	0 (0%)	0.241
	No	2 (100%)	16 (69.60%)	75 (77.30%)	69 (80.20%)	211 (76.20%)	16 (100%)	
Surgeons are not available	Yes	0 (0%)	8 (34.80%)	20 (20.60%)	28 (32.60%)	83 (30%)	6 (37.50%)	0.334
	No	2 (100%)	15 (65.20%)	77 (79.40%)	58 (67.40%)	194 (70%)	10 (62.50%)	

For knee injury as a reason for arthritis, no significant association was found between educational levels (p-value = 0.444). The absence of a substantial difference in awareness levels indicates a uniform understanding of the relationship between knee injury and arthritis among participants with different educational backgrounds. Regarding rheumatoid arthritis and the influence of growing old as reasons for knee arthritis, statistically significant associations were found (p-values = 0.012 and 0.002, respectively). Higher percentages of participants with postgraduate and bachelor degrees demonstrated awareness of these factors compared to those with lower educational levels. In exploring preventive measures for knee arthritis, participants with higher educational levels showed a statistically significant association with the awareness of losing weight (p-value = 0.014), exercise and motor activity (p-value = 0.038), topical cortisone injections (p-value = 0.035), drugs (p-value = 0.005), physical therapy (p-value = 0.012), and surgical intervention (p-value = 0.030).

For the purpose of knee replacement surgery, no statistically significant associations were found between educational levels and the awareness of relieving pain, increasing walking quality, the ability to exercise, and the ability to perform prayer. In examining factors influencing the decision to undergo knee replacement surgery, no significant associations were observed between educational levels and awareness of postoperative pain, general anesthesia and its complications, surgery not being helpful, and the availability of surgeons.

## Discussion

Osteoarthritis is a prevalent musculoskeletal disorder globally, contributing significantly to the burden of chronic conditions [2]. The knee joint is particularly susceptible to OA, and understanding community awareness and perceptions is crucial for developing targeted interventions [3]. This study explores the nuanced landscape of knee OA awareness and perceptions in the Northern Borders Region of Saudi Arabia, shedding light on demographic factors influencing these perspectives.

According to our results, gender-based differences in awareness were identified, particularly in recognizing obesity as a significant factor in knee OA. While 79.60% of males acknowledged obesity, 70.80% of females shared this awareness, indicating a slight but statistically significant difference (p = 0.021). This discrepancy may stem from varying sociocultural factors influencing health beliefs and behaviors, emphasizing the importance of tailored interventions for each gender [8,11].

Age-wise categorization highlighted a concentrated distribution within the 18-45 age groups, indicating the need for early interventions and preventive measures [13]. The recognition of obesity as a contributing factor showed significant age-related variations, with participants aged 46-65 exhibiting the highest awareness (78.70%, p = 0.040). Conversely, those under 18 demonstrated the lowest awareness (57.10%). This age-specific divergence underscores the necessity of age-tailored educational efforts to bridge knowledge gaps and instill preventive practices early in life [14,15].

Nationality-based analyses revealed a consistent understanding of obesity's role in knee OA across Saudi and non-Saudi participants. However, a significant association was found concerning knee injury as a reason for arthritis, with non-Saudi participants exhibiting higher awareness (58.30% vs. 29%, p = 0.028). This highlights the importance of considering cultural and contextual factors influencing awareness and perceptions of specific risk factors within diverse populations [12,16].

Educational levels played a pivotal role in shaping awareness and perceptions [16]. Participants with higher educational backgrounds demonstrated a more comprehensive understanding of various aspects, including rheumatoid arthritis and the influence of growing older as reasons for knee arthritis. Moreover, educational attainment significantly influenced awareness of preventive measures and treatment options. These findings emphasize the role of education in empowering individuals to comprehend the multifaceted nature of knee OA and make informed health-related decisions [12,14,15].

Comparing these findings with existing literature, the study corroborates the global trend that associates obesity with an increased risk of knee OA [11-16]. The high awareness levels regarding obesity as a risk factor align with studies conducted in different regions, emphasizing the universality of this perception [14]. However, gender-specific and age-related variations identified in this study contribute nuanced insights that may guide the tailoring of health education programs [17].

The gender-based differences in acknowledging obesity as a risk factor resonate with studies emphasizing gender-specific health beliefs and behaviors [11,13-15]. Sociocultural factors, role expectations, and access to health information may contribute to these disparities. Tailoring interventions to address these nuances is imperative for promoting equitable awareness and preventive practices [15].

Age-related variations in awareness align with studies indicating that older individuals may exhibit heightened awareness due to an increased likelihood of exposure to arthritis-related information over time [12,15]. However, the lower awareness among younger participants underscores the importance of early and targeted health education interventions to instill preventive measures and shape lifelong health behaviors [13].

The consistency in awareness across nationalities suggests that certain aspects of knee OA awareness may transcend cultural differences [18]. However, the higher awareness of knee injuries among non-Saudi participants underscores the importance of cultural sensitivity in health education initiatives, recognizing unique cultural perspectives that may shape awareness and perceptions [19].

Educational attainment as a determinant of awareness aligns with studies highlighting the positive correlation between education and health literacy [16]. The more nuanced understanding of preventive measures and treatment options among participants with higher educational backgrounds reinforces the need for accessible and tailored health information across diverse educational levels [16,19].

Implications and recommendations

The implications of this study are far-reaching, providing a foundation for targeted health education initiatives to enhance knee OA awareness and promote preventive practices in the Northern Borders Region, Saudi Arabia. Recognizing the demographic variations in awareness allows for the development of tailored interventions, acknowledging gender-specific, age-related, and educational nuances.

In light of gender-based differences, health campaigns could employ diverse communication channels and content strategies to ensure equitable access and impact. Incorporating sociocultural considerations may enhance the effectiveness of interventions in promoting a holistic understanding of knee OA [17,18].

Age-specific interventions, particularly targeting the younger population, are crucial for establishing preventive practices early in life [13,16]. Leveraging educational institutions, social media, and community engagement platforms can effectively disseminate information and shape positive health behaviors.

Cultural sensitivity should be central to health education initiatives, recognizing and respecting diverse perspectives on health and illness [12]. Incorporating cultural influencers, community leaders, and local healthcare providers in awareness campaigns can bridge cultural gaps and foster a more inclusive approach [18].

Educational programs need to be accessible and tailored to varying educational levels, employing clear and straightforward communication strategies. Leveraging digital platforms and community-based workshops can facilitate the widespread dissemination of information, ensuring that individuals across educational spectrums receive adequate and comprehensible health messages [14,15].

Study limitations and future directions

While this study contributes significantly to the understanding of knee OA awareness in the Northern Borders Region, certain limitations should be acknowledged. The cross-sectional design limits the establishment of causal relationships, warranting future longitudinal studies. Additionally, the study's reliance on self-reported data introduces the possibility of recall bias.

Future research endeavors could explore the effectiveness of targeted interventions developed based on the identified demographic variations. Longitudinal studies tracking changes in awareness and behaviors over time would provide insights into the sustained impact of health education initiatives.

## Conclusions

In conclusion, this study offers a comprehensive examination of knee osteoarthritis (OA) awareness and perceptions in the Northern Borders Region, Saudi Arabia. The findings highlight the influence of demographic factors, including region, gender, age, nationality, and educational levels, on community perspectives. By aligning these findings with existing literature, the study contributes insights for developing context-specific and tailored health education interventions. Recognizing and addressing the demographic nuances identified in this study will be instrumental in shaping effective strategies to enhance knee OA awareness and foster preventive practices in the community.
